# Development and Testing of an LED-Based Near-Infrared Sensor for Human Kidney Tumor Diagnostics

**DOI:** 10.3390/s17081914

**Published:** 2017-08-19

**Authors:** Andrey Bogomolov, Urszula Zabarylo, Dmitry Kirsanov, Valeria Belikova, Vladimir Ageev, Iskander Usenov, Vladislav Galyanin, Olaf Minet, Tatiana Sakharova, Georgy Danielyan, Elena Feliksberger, Viacheslav Artyushenko

**Affiliations:** 1 Art Photonics GmbH, Rudower Chaussee 46, 12489 Berlin, Germany; urszula.zabarylo@charite.de (U.Z.); vladimir240@mail.ru (V.A.); usenov@tu-berlin.de (I.U.); ef@artphotonics.de (E.F.); sa@artphotonics.com (V.A.); 2 Laboratory of Multivariate Analysis and Global Modeling, Samara State Technical University, Molodogvardeyskaya 244, 443100 Samara, Russia; valerya.belickova@yandex.ru (V.B.); v.galyanin@gmail.com (V.G.); 3 Medical Physics & Optical Diagnostics, CC6 Campus Benjamin Franklin, Charité Universitätsmedizin Berlin, Hindenburgdamm 30, 12203 Berlin, Germany; olaf.minet@charite.de; 4 Institute of Chemistry, St. Petersburg State University, Universitetskaya nab. 7/9, 199034 St. Petersburg, Russia; d.kirsanov@gmail.com; 5 Institute of Optics and Atomic Physics, Technical University of Berlin, Straße des 17. Juni 135, 10623 Berlin, Germany; 6 General Physics Institute of Russian Academy of Sciences, Vavilova 38, 119991 Moscow, Russia; ts@artphotonics.de (T.S.); gd@artphotonics.de (G.D.)

**Keywords:** tumor detection, kidney, fiber spectroscopy, optical sensor, near infrared, LED

## Abstract

Optical spectroscopy is increasingly used for cancer diagnostics. Tumor detection feasibility in human kidney samples using mid- and near-infrared (NIR) spectroscopy, fluorescence spectroscopy, and Raman spectroscopy has been reported (Artyushenko et al., Spectral fiber sensors for cancer diagnostics in vitro. In Proceedings of the European Conference on Biomedical Optics, Munich, Germany, 21–25 June 2015). In the present work, a simplification of the NIR spectroscopic analysis for cancer diagnostics was studied. The conventional high-resolution NIR spectroscopic method of kidney tumor diagnostics was replaced by a compact optical sensing device constructively represented by a set of four light-emitting diodes (LEDs) at selected wavelengths and one detecting photodiode. Two sensor prototypes were tested using 14 in vitro clinical samples of 7 different patients. Statistical data evaluation using principal component analysis (PCA) and partial least-squares discriminant analysis (PLS-DA) confirmed the general applicability of the LED-based sensing approach to kidney tumor detection. An additional validation of the results was performed by means of sample permutation.

## 1. Introduction

Cancer remains one of the main causes of mortality in Germany and worldwide [1]. To improve the situation, a larger number of diagnostic analyses should be performed at different stages of cancer medical handling, from preventive screening to treatment efficiency control. The implementation of this strategy results in a high demand for easy-to-handle, cheap diagnostic devices.

Malignant tumors are currently diagnosed using a number of methods, including non-invasive techniques such as computer tomography, ultrasound and X-ray scanning, as well as invasive procedures, including biopsies and diagnostic surgeries. Optical spectroscopy of the biological tissue, also called spectral histopathology [2], is a relatively new and highly promising approach to the non-invasive tumor diagnostics that has been actively investigated since the 1990s. Spectroscopic measurements are typically inexpensive and rapid, which makes the approach very well suited for diagnostic screening and routine prophylactic check-ups. As a promising method of tumor border detection, optical sensing capable of providing hundreds of real-time analyses presents a viable alternative to the traditional operative histopathology.

Neoplastic processes in a tissue inevitably result in its chemical and physical modification. These changes can be captured by the spectroscopic measurement of various effects accompanying the interaction of the tissue with an incident electromagnetic wave. Recent research results have confirmed the feasibility of differentiation between malignant and healthy tissues using fluorimetry and molecular spectroscopic techniques, that is, Raman scattering and infrared absorption or diffuse reflection [3,4]. The development of diagnostic spectroscopy results in a better understanding of chemical changes related to the pathological cell malfunction. In turn, in-depth knowledge of the disease’s biochemistry stimulates the development of new diagnostic methods and approaches.

Diffuse reflectance near-infrared (NIR) spectroscopy is one of the most promising methods for optical tissue analysis. Delivering a mixture of the scatter and absorbance information on the light-to-tissue interaction enables the detection of both molecular (related to the functional group vibrations) and morphological (due to the cell transformation) tissue changes, which makes it particularly useful for the oncology diagnostics of tumors. Successful NIR analysis applications for cancer detection in different tissues have been illustrated for the liver [5], the breast [6,7,8,9], the cervix [10], oral cancer [11,12,13], the prostate [14,15], the lung [16], gastric cancer [17] and the esophagus [18]. The most popular wavelength region, from 900 to 1700 nm, covered by the majority of modern NIR spectrometers includes intensive absorption bands of fat (lipids) and water known to be bright biomarkers of some tumor types, for example, of breast cancer [19]. It is important that the NIR light penetrates into the sample by up to 3 mm [20,21]; this depth cannot be reached by suitable spectroscopic techniques in other wavelength regions. Another important advantage of the NIR diagnostics of tissue tumors, compared to traditional methods, is the measurement flexibility due to the application of fiber-based light guides and probes, which is necessary under clinical conditions (e.g., during a surgery). The literature review revealed no data on kidney cancer diagnostics using fiber-based NIR spectroscopy.

Recent works have shown that high-resolution NIR spectroscopy can be replaced by optical sensing at a few specific wavelengths optimized for a particular application [22,23,24,25]. Technical downgrade does not necessarily result in an accuracy loss. Inexpensive sensors may perform as well as the respective full-scale spectroscopic techniques, or even better [20]. An inevitable loss of accuracy as a result of a much lower sensor resolution is compensated for by excluding less- and non-informative spectral regions (i.e., those containing irrelevant variance and noise) from consideration. The resulting simplification of the data and models tends to improve the model’s robustness and hence the reliability of a diagnosis.

In this study, a new tumor diagnostic sensor based on light-emitting diodes (LEDs) with emission wavelengths in the region of 900–1500 nm was developed and tested. To the best of our knowledge, the use of LED-based optical sensing for kidney cancer diagnostics has not yet been reported. Two sensor prototypes were implemented and tested for kidney cancer diagnostics using seven pairs each of tumor and normal in vitro clinical samples from different patients. Considering the novelty of the presented approach, the main analytical task of this study was to establish the fundamental feasibility of the LED-based optical diagnostics, that is, the sensor’s capability to capture cancer-related tissue variances at a few selected wavelengths. The measurements on real kidney samples are expected to be helpful for further sensor development.

## 2. Materials and Methods

### 2.1. Samples

NIR spectral and sensor measurements were obtained from unstained cryo biopsies of normal and tumor renal tissue in the Department of Urology at the Charité – Universitätsmedizin Berlin (Germany). Seven pairs each of normal and tumor samples from different patients were investigated after nephrectomy. The study complied with the Declaration of Helsinki. It was approved by the Ethics Committee of Charité, approval number EA1/134/12.

The sample thicknesses were typically from 5 to 10 mm, in accordance with common histopathological practice, as larger samples could not be shock-freezed as is required for clinical investigation. Histological classification was performed in accordance with the World Health Organization (WHO) criteria. The grading and staging were assigned following the rules of Fuhrman [26] and the International Union Against Cancer (UICC) [27], respectively. To prepare the tissue samples for analysis, they were thawed for 5 min at room temperature. It is common practice to use ex vivo samples at the early research stages, as in the present proof-of-concept study. Although the spectra of frozen/thawed biopsies can differ from real-life in vivo measurements, the possibility to transfer the diagnostic model was shown in a previous work on Raman spectroscopy of cancer [28].

**Figure 1:**
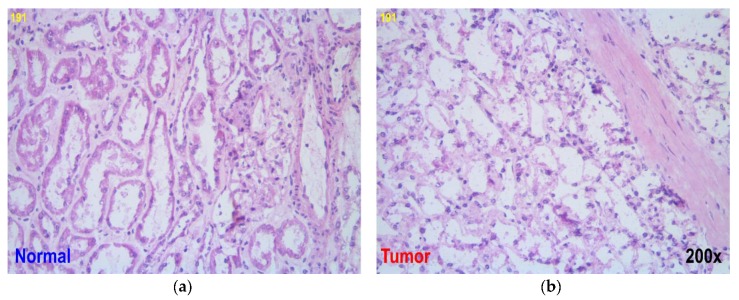
Biopsy microscope images of (**a**) normal and (**b**) kidney tumor tissue of patient 191 at 200× magnification.

The first series of samples for the spectroscopic study (Series A) included three pairs of biopsies corresponding to patient ID numbers 191, 194, and 198. Tumor samples in Series A belonged to grade 2, which designates (by the Fuhrman nuclear grading system [26]) slightly irregular contours of cells with diameters of about 15 µm and with nucleoli clearly observed under a microscope at a magnification of over 200× (Figure 1).

The next series (Series B) was measured using an improved sensor prototype and included four pairs of renal biopsies from patients 144, 149, 151 and 160; the respective tumor sample grades were 2, 1, 2, and 3. All the tumor samples in Series B belonged to the predominant clear cell renal cell carcinoma (cCRCC) sub-type.

**Figure 2:**
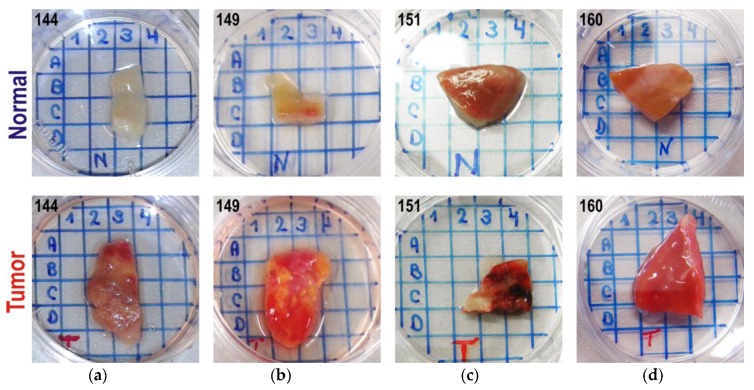
Renal biopsies of patients in Series B measurements: (**a**) 144, (**b**) 149, (**c**) 151, and (**d**) 160. Healthy and tumor tissues are marked as “N” and “T”, respectively. The grid period is 0.5 cm.

For the analysis, the tissue samples of renal biopsies were placed into separate 35 mm Petri dishes. To avoid any sample displacement during the measurement, the samples were glued to the bottom of the dish using a tissue-adhesive glue (TRUGLUE). A position-coding grid was drawn on the outer surface of the bottom of the dish (Figure 2). The measurements were performed in the center of each grid cell, which was entirely covered by the sample. An additional measurement of each sample was performed in a position corresponding to its geometrical center (the most remote from the edges).

Individual data were designated by the sample type (*T* and *N* for tumor and normal samples, respectively), the patient ID and the ordinal number of repeated spectral/sensor measurements. An additional index such as *C*3 or *B*2 in Series B denoted the measurement position on the grid (Figure 2); *Cn* was a special position in the sample center, irrelative to the grid. For example, *N*144*_Cn_*4 represents the fourth measurement in the center of the normal tissue sample for patient 144. The probe re-positioning was performed before each repeated measurement at the same position on the sample.

### 2.2. NIR Spectroscopy

Series A samples were initially studied using NIR spectroscopy in the region of 899.2–1721.4 nm. The NIR spectra were acquired using an Ocean Optics NIRQuest512 spectrometer (Ocean Optics, Inc., Dunedin, FL, USA) equipped with an InGaAs linear array detector with a resolution of about 3.1 nm and with a fiber-optic NIR reflectance probe by art photonics GmbH (Berlin, Germany). An LS-1 tungsten halogen light source by Ocean Optics was employed as a light source. The measurements were performed in the diffuse reflectance mode. The probe was fixed on a laboratory stand with a special clamp allowing for vertical movement. The head of the probe was equipped with a cylindrical stainless steel spacer to maintain a constant distance of 2 mm from the entrance of the optical fiber to the studied sample and to protect the measurement point from ambient light. The measurements were performed by bringing the tip of the spacer into immediate slight contact with the sample surface. Spectralon white diffuse reflectance standard (labsphere, Inc., North Sutton, NH, USA) was employed as a reference and was measured before each new biopsy sample. The spectra were acquired as an average of five scans of 150 ms each using OceanView instrumental software by Ocean Optics. The resulting dataset of Series A was a matrix formed by 41 spectra (in rows) recorded at 512 wavelengths. The data can be accessed at: https://tptcloud.com/data/view/3171.

### 2.3. Sensor Design and Data Acquisition

The main operating principle of any LED-based sensor is rapid sample scanning at several (usually from two to seven) different wavelengths. The LEDs perform an alternating illumination of the sample, and the remitted (back-scattered or reflected) light intensity is then detected by a photo diode. 

The LEDs in both constructed sensor prototypes operated in a pulse mode. Square 10 µs pulses at a frequency of 1000 Hz were used for the measurements. The pulses’ current intensity through the LEDs was 1 A. The operating conditions were chosen to provide spectral stability of the LEDs. Additionally, the pulse mode considerably slows down the degradation of LEDs, compared to continuous illumination. As the temperature noticeably affects the spectral properties of LEDs, this was controlled using a built-in semiconductor sensor. Throughout the whole experiment, the sensor’s internal temperature was maintained at 24 ± 1 °C (room temperature).

**Figure 3:**
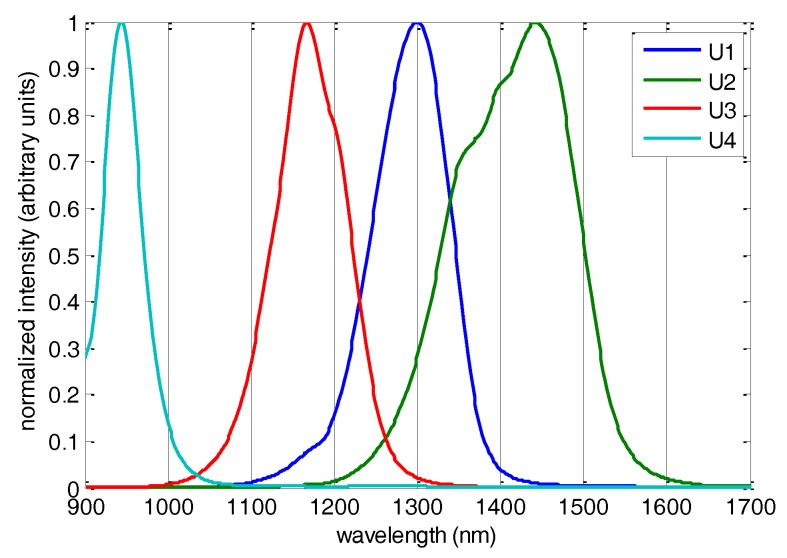
Normalized emission spectra of sensor light-emitting diodes (LEDs).

Four LEDs were used for the sensor construction with emission band maxima at 0.94 µm (sensor channel U4), 1.17 µm (U3), 1.30 µm (U1) and 1.44 µm (U2). The spectra of the chosen LEDs (Jenoptik, Jena, Germany) normalized to the emission intensity from 0 to 1 are presented in Figure 3.

**Figure 4:**
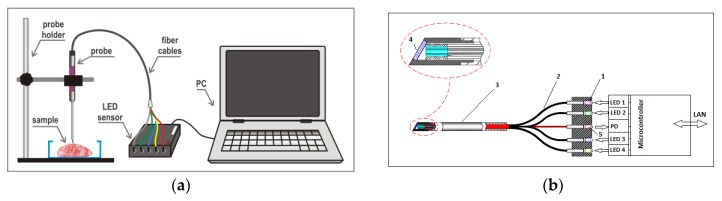
Probe construction and measurement setup. (**a**) Experimental setup, and (**b**) probe construction schematic: 1—light-emitting diodes (LEDs); 2—fiber cables; 3—stainless steel tube; 4—sapphire window (in Series A measurements, the probe tip had a right-angled shape and was not protected by a glass window); and 5—photodiode detector. (Reproduced with permission from authors [29]).

The sensors were equipped with 12 mm metal probes (Figure 4). In the experiment for Series A, the tip of the vertically mounted probe contacted an investigated sample through a thin (about 0.5 mm) quartz covering glass that protected the sample surface. In subsequent studies with an improved sensor (Series B), the probe was equipped with its own sapphire glass window located at Brewster’s angle (60.5°) relative to the probe axis (Figure 4b). During the measurements, the samples were touched by the probe’s sharp-angled tip only. In the latter case, therefore, the data was acquired at a distance of 3–4 mm above the tissue surface. The probe modification before Series B measurements were taken was implemented to eliminate the disadvantages observed for the initial sensor set-up: a non-optimal (too short) fibers-to-sample distance and strong specular reflection by the covering glass surface. Additionally, the built-in sapphire window protected the fibers and facilitated the probe cleaning.

The voltage data from the sensors was registered and saved by our own software written in LabView, version 14.0, by National Instruments (Austin, TX, USA).

The data can be accessed at: https://tptcloud.com/data/view/3179 (Series A); https://tptcloud.com/data/view/3180 (Series B).

### 2.4. Data Analysis Methods and Software

Multivariate data analysis was always prefaced by the mean centering of variables, that is, spectral wavelengths and individual sensor channels. This was the only preprocessing applied to the sensor data. The measurement *N194_5* was eliminated from the Series A sensor data as it was an evident outlier. Principal component analysis (PCA) [30] and partial least-squares discrimination analysis (PLS-DA) [31] were employed for the analysis of both the sensor data and the NIR spectra.

In the case of full-spectral NIR measurements, second derivatives of the spectral data were calculated using the algorithm by Savitzky-Golay [32] with a second-order polynomial and a smoothing window width of 25 points.

The following numbers were used as a statistical measure of the discrimination method’s performance: true positives (*TP*)—the number of correctly identified tumor samples; true negatives (*TN*)—the number of correctly identified healthy tissue samples; false positives (*FP*)—the number of healthy tissue samples incorrectly identified as a tumor; and false negatives (*FN*)—the number of tumor samples incorrectly identified as healthy. The above numbers were used to derive percent values for the sensitivity, *%Sn* = *TP*/(*TP* + *FN*), for the specificity, *%S*p = *TN*/(*FP* + *TN*), and for the accuracy, *%Ac* = (*TP* + *TN*)/(*TP* + *FP* + *TN* + *FN*) of the discrimination. The discriminant *Q*^2^ (*DQ*^2^) value was additionally used for the model’s performance assessment (Equation (1)). The *DQ*^2^ is an improved version of the conventional *Q*^2^ [33], which does not penalize the discrimination quality if correct predictions (*TP* or *TN*) fall out the interval formed by the calibration range [34] (from 0 to 1 in our case):

(1) $$DQ^{2}=1-\frac{\sum_{i:y_{i}=0\;\&\;{\hat{y}}_{i}>0}{\left(y_{i}-{\hat{y}}_{i}\right)}^{2}+\sum_{i:y_{i}=1\;\&\;{\hat{y}}_{i}<1}{\left(y_{i}-{\hat{y}}_{i}\right)}^{2}}{\sum_{i}{\left(y_{i}-\bar{y}\right)}^{2}}$$

where $y_{i}$ and ${\hat{y}}_{i}$ are the known and the predicted diagnosis, respectively, and $\bar{y}$ is the mean value of $y_{i}$ in the vector **y** of responses.

Data analysis was performed in MATLAB R2008b (The MathWorks Inc., Natick, MA, USA); PLS Toolbox, version 7.5 (Eigenvector Research Inc., Manson, WA, USA); and in the web-based chemometrics software TPT-cloud (www.tptcloud.com) by Global Modelling (Aalen, Germany), developed at Samara State Technical University (Samara, Russia).

### 2.5. Model Validation

Considering the limited availability of the biopsy material and the ethical aspects of its use, conclusions about the general suitability of the NIR sensor analysis for cancer diagnostics and, in particular, of four theoretically chosen LEDs are made on the basis of a very limited number of investigated patients. The necessary pairs of healthy and tumor biopsies could only be obtained from the whole kidney after nephrectomy, which constituted only about 30% of the total operation material (in accordance with the internal statistics of Charité). This was an additional complication limiting the sample availability.

In order to reduce the risk of overfitting caused by the limited experimental data volume, a thorough validation of the discrimination models was required. Three validation methods were applied to calculate the prediction statistics for each model: two types of cross-validation (CV) and a random-subset validation (RSV). The CV methods were full leave-one-out (LOO) CV and segmented CV procedures, for which the segments were formed by repeated measurements in different sample positions. (In Series A, for which arbitrary measurement positions were used, the segments were formed by six individual kidney samples.) In the third validation method, a random subset of measurements containing about 15% of the whole data set (the number of validation samples was chosen to be closest to this value) was excluded at the model building stage to be used for an independent prediction. To compensate for the random factor in the modeling statistics, the procedure was repeated 1000 times, and cumulative numbers of *TP*, *FP*, *TN*, and *FN* were used to calculate the *%Sn*, *%Sp*, and *%Ac* values. One-thousand iterations of the subset selection–modeling–validation cycle assured the convergence of the reported statistics to constant values independent of a particular subset. The optimal number of latent variables (LVs) to be retained in the PLS-DA models was determined on the basis of both CV methods.

The resulting discrimination models for the sensor data were additionally subjected to a permutation test (PT) [35]. The **y**-vector in the PT was randomized many times, before the following model building and validation. The PT passed if none of the random data arrangements resulted in better statistics than the original data. Typically, 50–100 PT trials were enough to reveal an overfitting. The average number of misclassified measurements (*NMC* = *FP* + *FN*) throughout all the RSV cycles was used as a PT merit function.

## 3. Results

### 3.1. NIR Spectroscopic Analysis and Sensor Simulation

**Figure 5:**
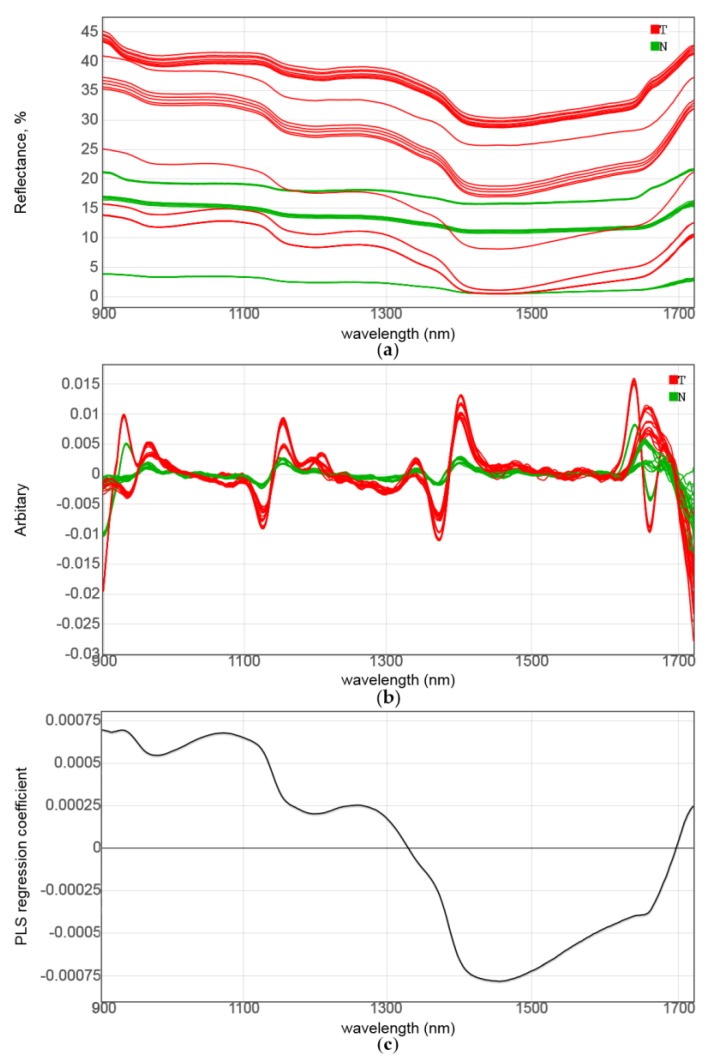
Analysis of near-infrared (NIR) spectrа in Series A: (**a**) raw spectra, (**b**) second-derivative spectra, and (**c**) partial least-squares discriminant analysis (PLS-DA) regression coefficients. Red and green spectra in (a,b) correspond to tumor tissue and normal tissue, respectively.

The NIR spectra of the Series A samples are presented in Figure 5a. Generally, high spectral background (reflectance percentage always remained below 50%, compared to the standard) was quite typical for the biological tissue. This accounted for a relatively deep penetration of light into the sample, where it could be absorbed or scattered and therefore lost for detection. The biopsy size or tissue-type differences, as well as seemingly insignificant variations of the sample position against the probe, may have resulted in a strong spectral variability. Thus, the reflectance percentage detected at 900 nm mostly varied between 45% and 15%, and in one instance, it even fell below 5%. Different effects of the background (i.e., offset shift) and the overall spectrum intensity (i.e., the distance between minimal and maximal values within a measurement) are clearly distinguishable. A strong susceptibility of the spectral background to various factors, resulting in both additive and multiplicative effects, is a well-known issue for the NIR measurements of highly scattering solids, for example, powders. The tumor samples exhibited generally higher offsets and a better contrast of the spectra, compared to the normal tissue, which could have been a consequence of their higher light-scattering ability. Nonetheless, the raw spectra do not have any features enabling reliable cancer identification with a naked eye.

The scatter-related variability can be mathematically eliminated using spectral derivatives. A data transformation into second derivatives (Figure 5b) revealed a number of variables enabling an unambiguous differentiation between healthy tissue and tumor tissue in the studied samples. Positive peaks in the second-derivative spectra correspond to the negative reflectance peaks of the components that could be hidden in the original data, although their derivative maxima could be shifted because of the presence of interfering components. The most intensive positive peak in the second-derivative data that is responsible for the diagnostics occurs at about 1400 nm. Two smaller but highly relevant peaks are located at 1155 and 965 nm. The discriminative ability of negative peaks in the middle part of the spectra should also not be neglected. In fact, the whole regions of 1100–1220 nm and 1350–1470 nm look very promising for cancer discrimination purposes. The former region is associated with C–H vibrations in the lipids, and the latter is known to include intensive second overtones of O–H bond vibrations in both lipids and water that may partially overlap [36]. It is remarkable that even the region of low absorbance at about 1300 nm exhibits some discriminative potential. In general, a visual inspection of the second-derivative spectra justified the theory-based choice of the LED set for a sensor construction that perfectly hits the regions of interest. 

The initial choice of LEDs was based on a priori knowledge of cancer biochemistry and optical properties of the biological tissue. Two illumination wavelengths, that is, those near 1.0 µm (about 1000 nm) and at 1.45 µm (1450 nm), were chosen in order to fit the corresponding water absorption bands’ maxima. One more LED with its emission maximum at 1.2 µm (1200 nm) was chosen to fit the lipid absorption band [37]. The ratio between water and lipids in the tissues was reported to be important for cancer detection with NIR instruments [19,38]. The fourth LED at 1.3 µm (1300 nm) was introduced into the system for scatter correction, as previous NIR measurements revealed high variability in the background signal. The spectral region around this wavelength was not densely “populated” in the NIR spectra.

**Figure 6:**
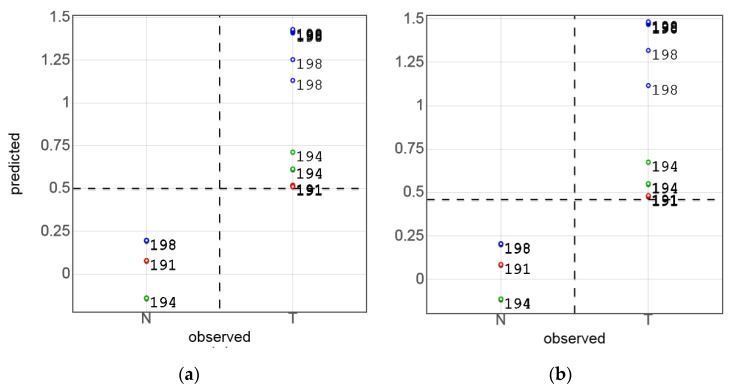
Partial least-squares discriminant analysis (PLS-DA)-predicted (sample-based segmented cross-validation (CV)) vs histologically observed tumor discrimination in Series A samples (patient ID numbers are indicated): (**a**) near-infrared (NIR) spectra, and (**b**) simulated sensor data.

The PLS-DA of the NIR spectra of Series A resulted in a full separation of the two sample classes (Table 1 and Figure 6a), even for the raw spectral data, using a model with two LVs. The measurements in different positions of the same biopsy tissue tended to form compact groups; therefore, all the samples were clearly distinguishable from each other, independently of the diagnosis. The tumor samples indicated higher diversity of measurements. The features of the model regression coefficient (Figure 5c), that is, smooth negative peaks at about 980, 1200 and 1450 nm, provided an additional statistical justification as to the importance of the chosen analytical regions for the LED sensor.

In the next development step, the full-spectrum data were used for sensor simulations. To simulate the sensor data, the spectral variables in the analytical regions were averaged using the real LED spectra (Figure 3) as weights. This operation resulted in a 41 (samples) by 4 (channels) matrix that was used to build another PLS-DA model for Series A. Despite the dramatic reduction of variables (from 512 in the NIR spectra to 4) the simulated sensor kept its diagnostic capability. Moreover, the predicted versus observed plots for the full and the reduced spectral data (Figure 6a,b) are very similar. Measurement points in the simulated sensor model (Figure 6b) have a somewhat higher scatter, in particular, for the tumor samples. As seven measurement points of the sample *T*191 fell below the standard discrimination threshold of 0.5, it had to be shifted to 0.47 in order to achieve full discrimination. Nevertheless, no misclassification was observed in either case, and the *DQ^2^* values (Table 1) revealed no significant statistical difference between the full-spectrum and simulated sensor models. 

**Figure 7:**
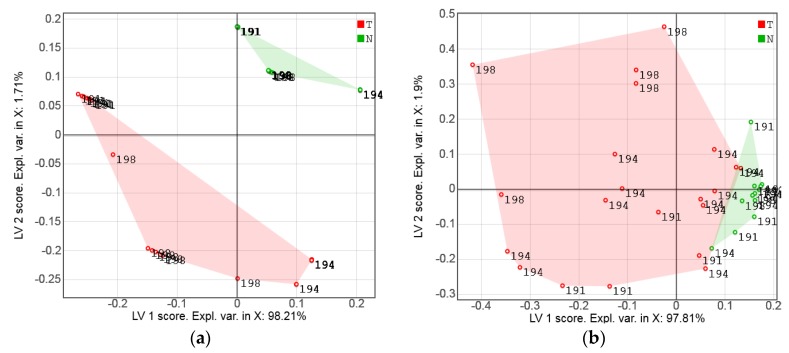
Partial least-squares discriminant analysis (PLS-DA) score plots: (**a**) simulated sensor data of Series A, and (**b**) sensor measurements of Series A. Tumor and normal tissue measurements are shown with red and green markers, respectively. The numbers designate patient IDs.

The class separation quality in the simulated data case can also be seen from the PLS-DA score plot in Figure 7a. The PLS-DA scores for the real sensor measurements of the same sample set (Series A), further discussed in Section 3.2, are presented in Figure 7b for comparison. Although the points are more strongly scattered in this case, a nearly full separation of the tumor and normal tissue classes is also observed.

The analysis of the NIR spectroscopic data presented here was performed for the primary purpose of sensor development. Full-spectrum measurements enabled the simulation of a four-channel sensor with theoretically chosen LEDs before its physical construction. The present discrimination modeling on the basis of poor experimental data including only a few patients was exploratory, but its results provide the necessary motivation for further sensor development. The practicable diagnostic model should be built at a later stage of the method development, using a large representative set of patients, and should then be permanently updated with new data. The representative set is expected to include all the main potential variability factors, such as the cancer type, grade and staging; and the age, gender, life-style, presence of others kidney diseases, and other patient-related factors. Clearly, the construction of such a model requires further extensive dedicated work, which is beyond the scope of this report.

### 3.2. Exploratory Analysis of Sensor Data

**Figure 8:**
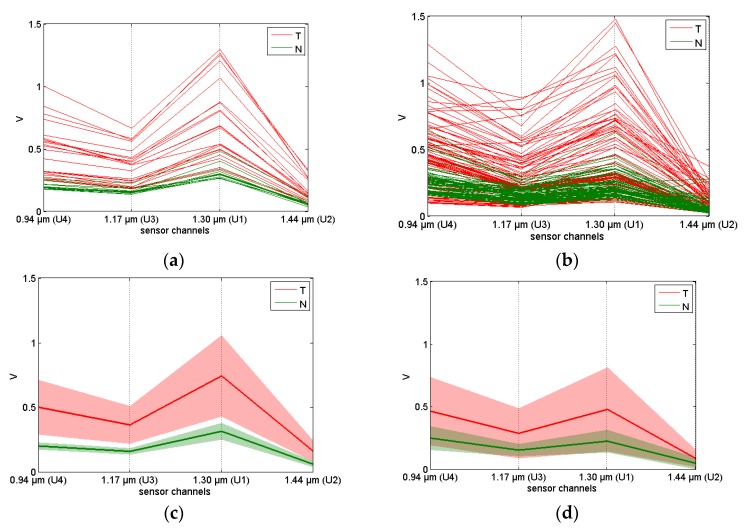
Sensor data: (**a**) Series A, (**b**) Series B, and (**c**,**d**) the data mean and standard deviation in Series A and B, respectively. Tumor and normal tissue measurements are shown with red and green lines, respectively. (Reproduced with permission from authors [29]).

As can be seen from Figure 8, two prototypes of the sensor (Section 2.2; Series A and B) yielded rather similar data. Figure 8c,d shows mean signals with the same shape, resembling those of the full-size NIR spectra, with a strong water absorption minimum at 1.44 µm. The channel U1 (1.30 µm) shows a particularly high variance. As a result of the different geometry of measurements (Section 2.3), Series B data generally have a lower intensity.

The averaged signals in Figure 8c,d imply that tumor tissues had generally higher values of detected signal intensity for all LEDs as a result of a greater light scattering, which is in a good agreement with the NIR spectra (Section 3.1). When compared with normal cells, cancer cells are known to have an increased DNA content, larger nuclear-cytoplasmic ratios and asymmetrical nuclear shapes. These differences are reflected in the NIR spectra because of the altering interaction of cells with the radiation; cCRCC tumor cells are typically arranged in compact alveolar, or acinar structures, such as nests, sheets, etc., and they have a clear cytoplasm. The diameter of cancer cell nuclei can reach 20 µm, while it is around 5–10 µm for normal cells. In these circumstances, the most efficient light scatterers are generally cell nuclei [39]. The backscattered light intensity at the chosen wavelength depends on the optical properties of the tissue. These properties are strongly related to the size distribution of the cells and organelles responsible for the differentiation between the normal and abnormal tissue. Significant biochemical and morphological modification of the tumor-affected tissue (Figure 1) seems to be the major reason for its higher scattering coefficient [40].

A higher homogeneity of healthy kidney tissues results in a much lower sensor signal variance for corresponding samples. A rich lipid content in RCCs yields a characteristic golden-yellow appearance of the samples. A varying degree of cystic degeneration, blood particles, calcification and necrosis [41] leads to inhomogeneous cut surface (e.g., Figure 2). The possibility of distinguishing between normal and tumor tissue samples is essentially based on the backscattered light intensity. This is confirmed by the fact that the application of any filtering procedure intended for scatter correction (e.g., standard normal variate (SNV)) seriously deteriorates the discrimination power of the mathematical model. This was observed when using one of the channels as an internal reference. Thus, the data sets for Series A and B were analyzed in a multivariate mode without any scatter-correcting preprocessing.

The PLS-DA score plot (Figure 7) is a convenient way to visualize the “strength” of the cancer discrimination. In Series A, the tumor/normal class separation in the plane of the two first LVs is almost complete (Figure 7b). A few measurement points showing discrimination ambiguity problems were related to the spectral similarity of two different samples from patient 194. This could be explained by the high content of fat in both the tumor and normal samples of this patient. The bright golden-yellow color of the tumor sample *T*194 was clearly due to intracellular lipid accumulation, while the normal sample *N*194 might have been a cut of the adipose capsule of the kidney (or perinephric fat), which is a structure between the renal fascia and the renal capsule. In spite of a reasonably strong variance in the measurements of tumor samples observed in the factor space of the PLS-DA models, the overall tendency looks very promising. While none of the individual channels in Figure 8 can allow for a reliable discrimination of the samples, multivariate data processing yielded rather convincing results. 

**Figure 9:**
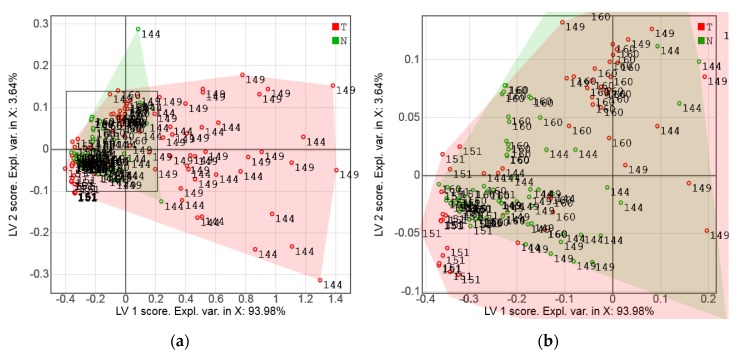
Score plot of the first two latent variables (LV2 vs LV1) for a principal component analysis (PCA) model for the sensor data of Series B: (**a**) full area, and (**b**) magnification of selected area in (**a**). Tumor and normal tissue measurements are shown with red and green markers, respectively. The numbers designate patient IDs.

The class separation was less successful for the Series B data. Prior to the PLS-DA modeling, the data’s internal structure was investigated by PCA in order to detect outliers and non-typical measurements. Optical properties of certain individual samples resulted in some discrimination problems. For example, sample *T*151 yielded a very compact cluster of points at the left side of LV1, while the tumor was mainly associated with the positive LV direction. Figure 2a shows that these two particular samples from patient 151 had very different visual appearances. The investigation of some specific locations on the samples revealed further inconsistencies. Thus, the PCA score plot (Figure 9) shows that all *Cn* measurements of *T*144 exhibited similar trends to the tumor sample *T*151 (located in the healthy group), although other measuring locations of *T*144 appeared in the red zone. On the other hand, *Cn* measurements of *N*144 (normal sample of this patient) were false positive outliers. The PLS-DA modeling statistics (Table 1) could be improved significantly by the elimination of the only sample *T*151 and two measurement positions *N*144*_Cn* and *T*144*_Cn* (about 18% of the data).

### 3.3. PLS-DA of Sensor Data and Model Validation

Unlike PCA, the PLS-DA is a supervised classification method using the factor space optimized for a particular discrimination task. The calibration and validation statistics of PLS-DA models for Series B are given in Table 1. No large discrepancy between the calibration and different validation statistics is observed. As expected, the full CV was generally more optimistic, but changing the validation method to segmented CV did not result in any dramatic consequences for the models. The segmented CV with the segments formed by measurement positions in Series B was the most straightforward CV strategy in the present research. Repeated measurements in the same sample position produced similar spectra or sensor data; therefore, these should always be simultaneously excluded for every CV iteration. The sample-based segmented CV was perhaps too crude because of a high risk of model bias, when one of three available tumor or normal samples was eliminated from the training dataset.

RSV is another viable option to validate multivariate models built on relatively small datasets. The subset volume of 15% was a reasonable trade-off between the representativeness of the residual training set and the statistical reliability of the prediction. It has been verified that varying the subset size between 10% and 20% does not affect the modeling statistics significantly. RSV combines the advantages of independent validation (although it is not strictly independent because of the repeat-and-average approach) with efficient data use, as in CV. RSV statistics take an intermediate position between the LOO and segmented CV approaches. It is remarkable that for the most representative refined sensor model, Bs140, the RSV metrics were virtually the same as for LOO CV (Table 1).

**Figure 10:**
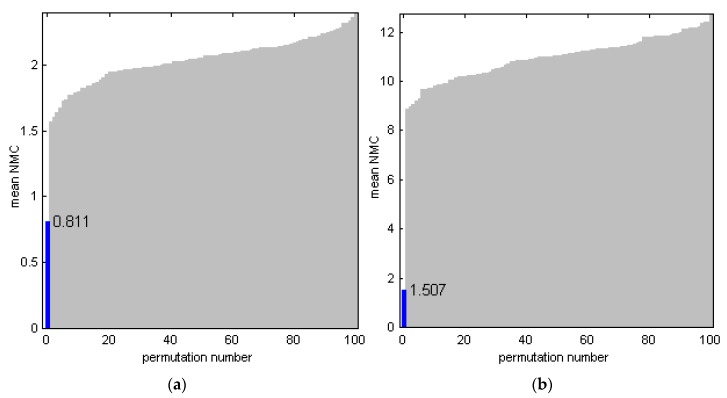
Permutation tests of partial least-squares discriminant analysis (PLS-DA) models for sensor measurements: (**a**) Series A, and (**b**) refined Series B (As33 and Bs140 models in Table 1, respectively). Validation with a random 15% subset at 1000 iterations was used; sorted mean number of misclassified measurements (*NMC*) metrics are presented. Blue bar at zero permutation number corresponds to original non-permuted data.

The models for the sensor data As33 and Bs140 successfully passed PTs (Section 2.5). A large difference between the *NMC* statistics for the original and any randomized order of the samples in the **y**-vector of responses (Figure 10) additionally confirmed the predictive capabilities of the respective PLS-DA models. A high efficiency of the **y**-permutation test to protect against chance correlation has been illustrated in [42].

The evident consistency of different validation and calibration statistics presented in Table 1 as well as the PT results provide convincing proof that our preliminary discrimination models reflect the real sensor capabilities of tumor diagnostics. The moderate complexity of the models (two LVs) is further evidence against any significant overfitting.

A thorough validation, including full, segmented and random-subset CVs, as well as randomization tests, are recommended in any cases for which important conclusions depend on calibration or discrimination models built on limited experimental material.

As a general conclusion from the experimental analysis of the kidney samples, it can be stated that the LED-based sensing approach developed in the present work is capable of discriminating between tumor and healthy tissues. Taking into account the data diversity in Series B, it can be taken as a reasonably good first estimate of the real-life applicability of the sensor. The technical quality of the method is additionally confirmed by a good reproducibility of the measurements in individual samples and in particular sample sites.

## 4. Discussion and Conclusions

LED-based optical sensor technology has already been shown to be suitable for the production of miniaturized chemical sensors and optical diagnostic devices (e.g., pulse oximetry and detection of oral cancer [43]). The presented combination of LEDs with simple photodiode detection provides a good alternative to full-sized spectroscopic devices and allows for a fine tuning of the analysis to the requirements of a particular practical task. Another method advantage is the possibility of miniaturization, which is especially important in the context of real-time medical diagnostics. Distinguishing between tumor and non-tumor tissues using LED-based sensors is a new approach, and much work remains to be done. A significant technical simplification of the LED-based sensing, compared to the conventional full-range spectroscopy, should result in a dramatic reduction of the device’s purchase and maintenance prices. Additionally, LED sensors are portable and do not require the permanent availability of a computer and power supply. The appearance of a budget tool for cancer diagnostics, and hence, the growing total number of analyses are expected to have a positive impact on human health worldwide. The choice between a spectrometer and an LED-based sensor does not necessarily indicate a trade-off between the analysis price and reliability. Using a limited number of wavelengths rather than the full spectrum makes it easier to focus on the most informative signals, therefore avoiding the areas of noise and irrelevant variance. This concept is particularly useful for the investigation of complex biological objects.

This study provides a proof-of-concept demonstration of the LED-based sensor for tumor discrimination. Only four LEDs with emission wavelengths in the NIR range were employed. Further dedicated studies are needed to yield the next sensor prototype. For example, examinations of various organs, the selection of optimal wavelengths, and data processing methods must be addressed. The presented technology of diffuse reflectance NIR sensing for tumor diagnostics can be extended to other tissue and cancer types. To provide the optimal sensor performance, the working LED set, that is, the LED number and wavelengths, should be adapted to each case individually to take respective biomarkers and optical properties of the tissue into account. The experimental and data analysis approaches suggested here can be used to minimize the number of clinical measurements during the development stage.

The main disadvantage of the LED-based sensor in its present form is a very high measurement variance in cancer tissue samples (Figure 9). The main requirement for the sensor improvement is therefore to increase the accuracy and specificity of detection, as *FN* are unacceptable outcomes in oncological diagnostics. Although the sensor performance demonstrated by the present prototype should be further improved, the results reported here can be considered as a successful feasibility study of an LED-based NIR sensor for cancer diagnostics aimed at in vivo applications. The sensor’s general sensitivity to the kidney tumors, illustrated here, provides the necessary motivation for further development of the method.

The directions for the further improvement of the sensor are (1) LED number and wavelength optimization, (2) optimization of the measurement conditions (distance, angle, light intensity, spectral reference, probe construction, elimination of environmental effects, etc.), and (3) data processing optimization. These improvements represent an important part of the future work.

## Figures and Tables

**Table 1:** Partial least-squares discriminant analysis (PLS-DA) calibration and validation statistics of tumor detection in Series A and B; two latent variables (LVs) were used in all models.

Data	*DQ^2^*	*TP*	*FP*	*TN*	*FN*	*%Sn*	*%Sp*	*%Ac*
	Calibration ^1^							
An41 ^2^	0.932	21	0	20	0	100.0	100.0	100.0
As41 ^3^	0.917	21	0	20	0	100.0	100.0	100.0
As33 ^4^	0.437	19	1	11	2	90.5	91.7	90.9
Bs170 ^5^	0.181	62	5	70	33	65.3	93.3	77.6
Bs140 ^6^	0.500	64	3	67	6	91.4	95.7	93.6
	Full (leave-one-out) cross-validation							
An41	0.920	21	0	20	0	100.0	100.0	100.0
As41	0.902	21	0	20	0	100.0	100.0	100.0
As33	0.358	18	1	11	3	85.7	91.7	87.9
Bs170	0.153	59	7	68	36	62.1	90.7	74.7
Bs140	0.478	63	3	67	7	90.0	95.7	92.9
	Segmented cross-validation ^7^							
An41	0.574	21	0	20	0	100.0	100.0	100.0
As41	0.488	14	0	20	7	66.7	100.0	82.9
As33	0.051	13	3	9	8	61.9	75.0	66.7
Bs170	0.068	51	7	68	44	53.7	90.7	70.0
Bs140	0.416	61	3	67	9	87.1	95.7	91.4
	Random-subset validation ^8^							
An41	0.916					100.0	100.0	100.0
As41	0.899					100.0	100.0	100.0
As33	0.345					82.4	86.1	83.8
Bs170	0.149					60.2	91.0	74.8
Bs140	0.480					89.9	95.7	92.8

^1^ Both training and validation of the model were performed using the full dataset. ^2^ Near-infrared (NIR) spectra of Series A. ^3^ Simulated sensor data in Series A. ^4^ Sensor measurements in Series A. ^5^ Sensor measurements of full data in Series B. ^6^ Sensor measurements of Series B without sample *T*151 or positions *N*144*_Cn* and *T*144*_Cn*. ^7^ Segments were formed by samples (Series A) or measurement positions (Series B). ^8^ Subset (15%) of the full data at 1000 iterations.
